# Plasmonic nanopatch array for optical integrated circuit applications

**DOI:** 10.1038/srep03172

**Published:** 2013-11-08

**Authors:** Shi-Wei Qu, Zai-Ping Nie

**Affiliations:** 1 School of Electronic Engineering University of Electronic Science and Technology of China (UESTC) 2006 Xiyuan Avenue, Western High-Tech Zone Chengdu 611731, China

## Abstract

Future plasmonic integrated circuits with the capability of extremely high-speed data processing at optical frequencies will be dominated by the efficient optical emission (excitation) from (of) plasmonic waveguides. Towards this goal, plasmonic nanoantennas, currently a hot topic in the field of plasmonics, have potential to bridge the mismatch between the wave vector of free-space photonics and that of the guided plasmonics. To manipulate light at will, plasmonic nanoantenna arrays will definitely be more efficient than isolated nanoantennas. In this article, the concepts of microwave antenna arrays are applied to efficiently convert plasmonic waves in the plasmonic waveguides into free-space optical waves or vice versa. The proposed plasmonic nanoantenna array, with nanopatch antennas and a coupled wedge plasmon waveguide, can also act as an efficient spectrometer to project different wavelengths into different directions, or as a spatial filter to absorb a specific wavelength at a specified incident angle.

Ultra-fast communication systems with extremely huge data rate are always pursued for a variety of applications. However, the data rate of current systems with traditional signal transmission mechanism based on electronics is significantly restricted due to the finite speed of electrons in metal. Therefore, fully optical integration scheme is a hopeful alternative to the present electronic counterpart^1^. However, one of the most troublesome problems confronted by optical integrated circuits (ICs) is the bulky dimensions of traditional photonic devices due to the diffraction limitation, e.g., lens, optical fiber, and optical coupler, which makes an intense integration of optical circuits impossible in practice. Recently, plenty of researches on plasmonics provide an effective way to solve it, in which the optical fields are highly confined beyond the diffraction limitation^2^,^3^,^4^. Essentially, surface plasmons are the collective resonance of electrons at the metal-dielectric interface and then the optical power is transformed into surface waves propagating along the interface. In the past several years, many kinds of plasmonic waveguides were proposed for either efficient transmission of optical signals or high confinement of optical fields, e.g., channel plasmon waveguides (CPPs)^5^,^6^, metallic nanowires^7^, metal nanoparticle chains^8^, strips^2^, dielectric loaded plasmonic waveguides^9^, wedges^10^,^11^, nanoslots^12^, and hybrid plasmonic waveguides^13^. Meanwhile, some optical devices were also investigated based on the proposed plasmonic waveguides, e.g., plasmonic coupler^14^, splitter, resonant ring^5^, lens^15^, optical logic gate^16^, and Bragg grating filter^17^, achieving many pathbreaking results toward the optical ICs.

Concept of plasmonic nanoantennas, as one of the most important achievements, was firstly proposed for optical field enhancement. Since then, many researchers concentrated on developing different kinds of plasmonic nanoantennas, e.g., bowtie antennas^18^, monopoles^19^, dipoles^20^, particles^21^, grating antennas^22^, coupled nanoholes^23^, nanowires^24^, and Yagi nanoantennas^25^ for different applications from light emissions or excitations^26^ to photonvoltaics^27^, biomedical science^28^, optical wireless nanolink^25^,^29^ etc. However, patch antennas, quite popular at microwave frequencies due to very simple structure, were scarcely reported at optical wavelengths. The nanopatch antenna was firstly proposed by R. Esteban for single photon emission^30^, then followed by other works on the nanopatch antenna elements^31^,^32^ and arrays^33^,^34^ around the world. Unfortunately, the reported nanopatch arrays, all excited by free-space optical beams but without any power guiding waveguides^33^,^34^, are still far from the optical IC applications. Meanwhile, approaches to design microwave antenna arrays often confront many fatal problems at optical wavelengths, especially quite low efficiency due to the lossy plasmonic waveguides.

We report herein a nanopatch array excited by a coupled wedge plasmon waveguide (CWPWG) for the optical IC applications. It is capable of highly directive emission and has potential applications for spectrometers or spatial colour filters. Meanwhile, the design can be applied to other wavelengths by properly scaling the nanopatch antennas and the CWPWG based on the dispersion relations of the employed metal^35^. The key concept here is to couple the CWPWG guided plasmonics into the resonant nanopatch antennas and then convert them into free-space radiation. Compared with the aforementioned nanoantenna arrays, the proposed one presents many merits, e.g., simple structures, significantly improved bandwidth, and more importantly, direct connections to the optical subwavelength waveguide which is essential to realization of the optical ICs.

## Results

### Plasmonic nanopatch array

**Figure 1:**
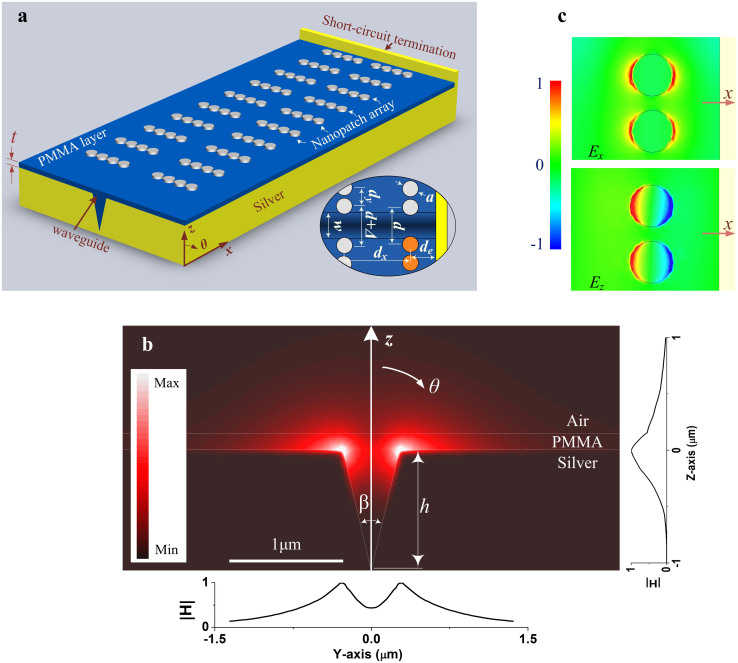
Nanopatch array with the CWPWG. (a) Schematic diagram of the proposed array with 8 × 10 plasmonic nanopatch antennas. The inset in the lower-right corner shows dimensions and relative positions of the nanopatch antennas. *t* = 150, *h* = 1080, *a* = 340, *d_y_* = 400, *d_x_* = 1250, *w* = 500, *d_e_* = 485, *d* = 700, *Δ* = 10 (in nm) and *β* = 26°. (b) Magnetic field distributions in a cross section of the CWPWG. Normalized amplitude of the magnetic fields along the z axis is given in the inset at the right-hand side of the figure, and that along the line orthogonal to the CWPWG axis, 50nm above the top surface of the silver film, is at lower side. The fields are concentrated in an area close to the groove aperture. Therefore, as a nanopatch antenna is placed adjecant to the groove, it will be excited with a coupling coefficient inversely proportional to its distance to the groove. (c) The x- and z-components of the electric fields on the top layer of the PMMA, i.e., *E_x_* and *E_z_*. Two nanopatches correspond to those in the lower-right corner of the inset of Figure 1a, as shown in orange.

Figure 1a presents geometry of the proposed array, and 10 × 8 circular nanopatch antennas made of silver are symmetrically arranged on both sides of a CWPWG which is constructed on a silver film. The groove and the space between the nanopatch layer and the silver film are filled with polymethylmethacrylate (PMMA), with an effective index of 1.49^9^. Dispersive properties of silver can be described by the Drude permittivity model described by equation (1)

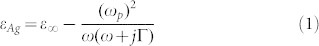
where ε_∞_ = 5, the plasma frequency ω_p_ = 2π × 2.133 × 10^15^ rad/s, and the collision frequency Γ = 1.12 × 10^14^ Hz, to fit the experimental value of −116.38 − *j*11.1 at the wavelength of 1.55 μm^36^.

Fields of the air-filled CPPs^2^,^3^,^4^ are confined at the bottom of the groove, therefore difficult to be coupled into a fundamental mode nanopatch antenna. Comparatively, the PMMA loaded CWPWG presents a symmetric hybrid s-polarized mode with a larger mode size, as shown in Figure 1b, whose magnetic fields are mainly concentrated in a small area close to the groove aperture, a large part of them within the PMMA and the left within the air and silver. The magnetic fields exponentially decay in +z and ±y directions far away from the groove aperture, as shown by two insets in Figure 1b. Meanwhile, different from the fundamental CPP mode, the fields are also decayed into the groove. Due to the low propagation loss, the field decay in the propagating direction along the CWPWG will be much slower. Interestingly, the mode size is dominated by the flare angle *β* of the groove, instead of thickness of the PMMA layer, but wave number of the propagating mode in the CWPWG depends on both of them. (See Supplementary Figs. S1 and S2 for the electric field distributions, influences of the groove flare angle *β* and thickness *t* of the PMMA layer). Unlike for field confinement purposes in many previous literatures^2^,^3^,^4^, the scenario of less confined fields is of benefit to nanopatch array design herein, because the optical power will be easier to be coupled onto the nanopatch antennas. Meanwhile, the PMMA loaded CWPWG is more practical under considerations of metal oxidization, availability of multi-layered optical devices etc, except for purposes of supporting the nanopatch antennas. At the far end of the CWPWG, a silver step with a height of 500 nm acts as a short-circuit termination to avoid direct emission of a physically open CWPWG to +x direction, which will result in large undesired parasitical beams. (For the CWPWG, a physically open end is far from an electrically open-circuit termination in terms of plasmonic wave behavior, and the open circuit is actually quite difficult to realize at optical frequencies.) Then, standing plasmonic waves are formed along the CWPWG.

As the nanopatch antennas operated in the fundamental mode are arranged along the y axis by a distance *d_e_* to the short-circuit termination, symmetrically with respect to the CWPWG, they can be excited by the electric fields of the plasmonic standing waves in the CWPWG. Comparatively, previously reported nanopatch antennas with quantum dots located at their center^30^,^32^ are actually operated in higher order mode. The coupling coefficient can be controlled by a distance *d* between the nanopatches on both sides of the groove. For an x-direction polarized emission beam, every row of nanopatches should be arranged at the node of *E_z_* or at the antinode of *E_x_* for an efficient inductive or magnetic coupling (*E_z_* and *E_x_* are the z- and x-components of the electric fields, respectively). In Figure 1c, *E_z_* is confined under the nanopatch antenna in the fundamental mode, forming a resonant cavity between the nanopatch and the silver film, while *E_x_* directly contributes the emission into the upper half space. Meanwhile, intensity of the fields under the nanopatch closer to the groove (upper one) is slightly stronger than the one farther (lower one), because of the decayed field intensity from the central axis of the groove to the ±y direction. Albeit similar to microwave patch antennas in geometry, the operating principle is quite different due to the plasmonic effects of metal at optical wavelengths. Resonant diameter *a* of the plasmonic nanopatch is only around one fifth of the operating wavelength in free space, and there are also parallel components of the electric fields to the patch surface due to the plasmonic effects. Meanwhile, compared to the nanopatch antenna without the silver film below^31^, cross talk between the fields on the nanopatch and on the silver film will be of benefit to array performances in this work. First it will concentrate more optical power into the PMMA, consequently reducing the power loss in silver and improving the array efficiency. Second it also changes the emission mode of the nanopatch by introducing an image beneath the silver film^37^, causing an emission beam towards +z direction, instead of multi-directional beams of the isolated nanopatches^30^,^32^.

The redirection of emission can be quantified by the expression of directivity, defined by the ratio of the power per unit solid angle *P*(*θ*, *ϕ*) emitted in a given direction to that emitted by an isotropic source of equal total radiated power, as given by equation (2)^37^

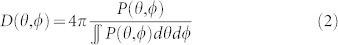
From emission point of view, as the plasmonic waves are injected into the CWPWG, a small part of them is coupled in turn to the first, the second row of nanopatch antennas and so on in Figure 1a. A gradually larger coupling coefficient is required for an optimized distribution of the electric fields on all rows of the nanopatches, and therefore a small difference *Δ* = 10nm is set between two neighboring rows for a tradeoff between a maximum peak directivity, a larger beam shifting range and a broader spectral width.

### Optical property of the plasmonic nanopatch array

**Figure 2:**
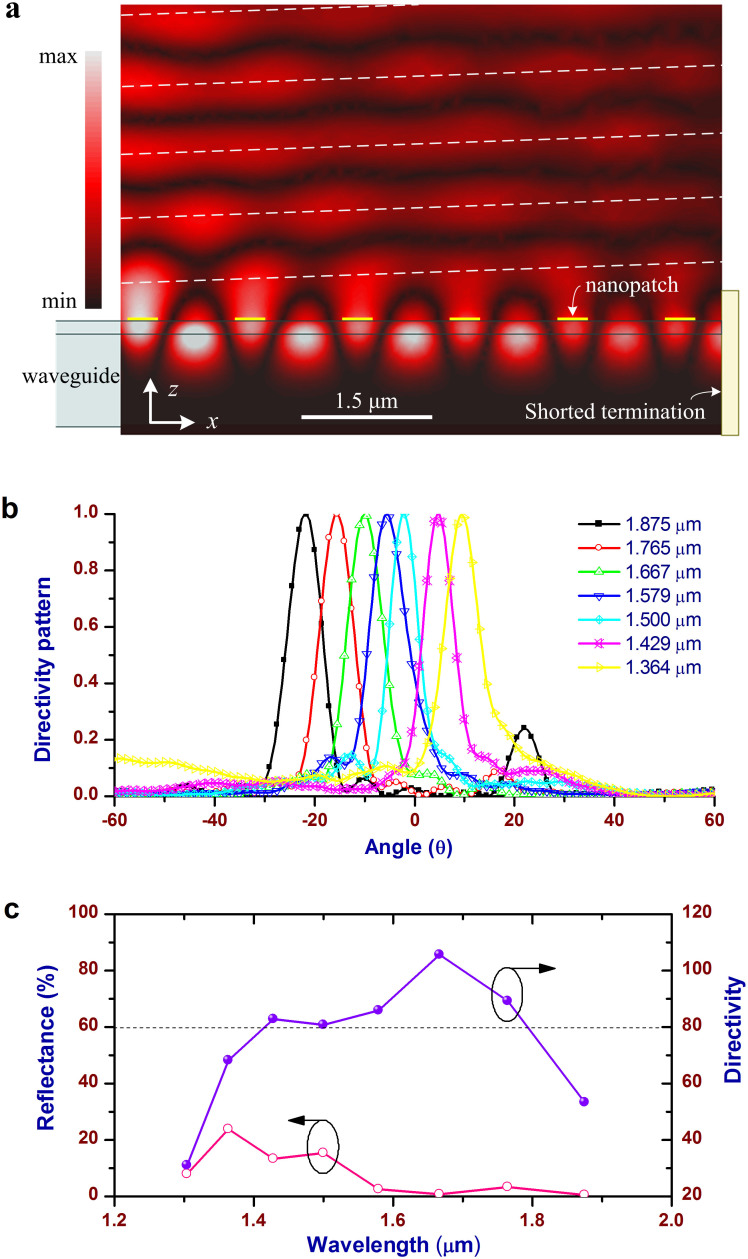
Results of the proposed nanopatch array. (a) Magnetic field distributions in the central plane at the wavelength of 1.5 μm. The central plane is the one parallel to the xz plane and passing through the bottom line of the groove, with respect to which the proposed array is symmetric. Power emissions by the nanopatch antennas are clearly seen. The field intensity is gradually decreased along the x axis mainly due to the power emission. At the end of the CWPWG, the left small part of power (less than 3% of the excitation power for the array with 10 rows of nanopatch antennas) is terminated to avoid direct emission towards the x-axis direction. (b) Normalized directivity patterns of the nanopatch array at different wavelengths. For wavelengths from 1.364 to 1.875 μm, the beam is pointed from 9.5° to -22°. The average beam shifting gredient, beam shifting range over wavelength bandwidth, is around 6.2°/100 nm. (c) Reflectance observed in a reference plane of the CWPWG, 1.6 μm far from the first row of nanopatch antennas, and the peak directivity of the proposed nanopatch array. The maximum reflectance of around 23% occurs at 1.364 μm, and the reflectance is below 3.5% as the wavelength is over 1.579 μm. The peak directivity is above 80 from 1.429 to 1.765 μm, with a maximum of 105.7 at 1.667 μm.

Figure 2a shows magnitude distribution of the magnetic fields in the central plane of the structure. Above statements of the optical power propagated along the CWPWG and emitted into free space can be clearly proved, and only a very small part of power is reflected back by the termination (See Supplementary Figs. S3 and S7 for the field distributions and animations at all wavelengths). Figure 2b gives the normalized directivity patterns of the proposed nanopatch array at different wavelengths, and the emission beam is pointed from 9.5° to −22° as the wavelength is increased from 1.364 to 1.875 μm. Therefore, as the first merit, the proposed array with very compact dimensions can replace traditional bulky photonic spectrometers or spatial filters widely applied in optics. For shorter wavelengths outside the operating spectral width, the beam cannot be scanned any more, while for longer wavelengths the peak directivity will be sharply reduced. In Figure 2c, the peak directivity and power reflectance of the nanopatch array are presented. As the second merit, there is a broad spectral width from 1.429 to 1.765 μm in which the peak directivity is over 80 with a maximum of 105.7 at the wavelength of 1.667 μm, indicating its capability to handle the optical beams with a high spatial resolution as well as to intercept more free-space optical power in an excitation mode (See Supplementary Fig. S8 for results obtained by the finite integration technique).

**Figure 3:**
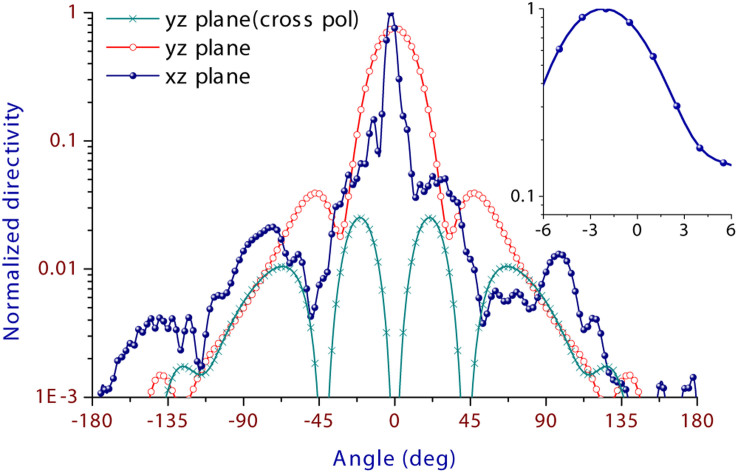
Two-dimensional (2D) directivity patterns at 1.5 μm. In the xz plane, the principle polarization is along θ direction, while it is along *ϕ* direction in the yz plane. Meanwhile, magnitude of the cross polarization in the yz plane is only 2.5% of the peak directivity, and that in the xz plane is even below 1‰, indicating excellent linearly polarized characteristics of the nanopatch array. The inset shows the beam at 1.5 μm is pointed at *θ* = -2°.

The larger reflectance at shorter wavelengths, with a maximum 23% at 1.364 μm, is attributed to higher mismatching caused by nonresonance of the nanopatch antennas at these wavelengths. Its functional spectral width is collectively determined by the amplitude of parasitical beams, the reflectance by the nanopatch array and the drop of peak directivity. In terms of a reflectance of below 25%, a parasitical beam amplitude of below 25% relative to the main beam, and a peak directivity of over 80, the spectral width can be over 340 nm. The array efficiency, defined by the total emitted power into free space over the total accepted power, is in a range of 61% ~ 75.4% for all wavelengths from 1.364 to 1.875 μm (See Supplementary Fig. S4). Figure 3 shows the xz- and yz-plane normalized directivity patterns at 1.5 μm to show details of the array beam, and only a negligible part of optical power is emitted into the lower half space (|θ| > 90°) (See Supplementary Figs. S5 and S9 for 3D directivity patterns at all wavelengths). In the yz plane, the parasitic beams are as small as 4% relative to the main beam due to the symmetric array structure. In the xz plane, they are a little higher, but below 17% relative to the main beam.

**Figure 4:**
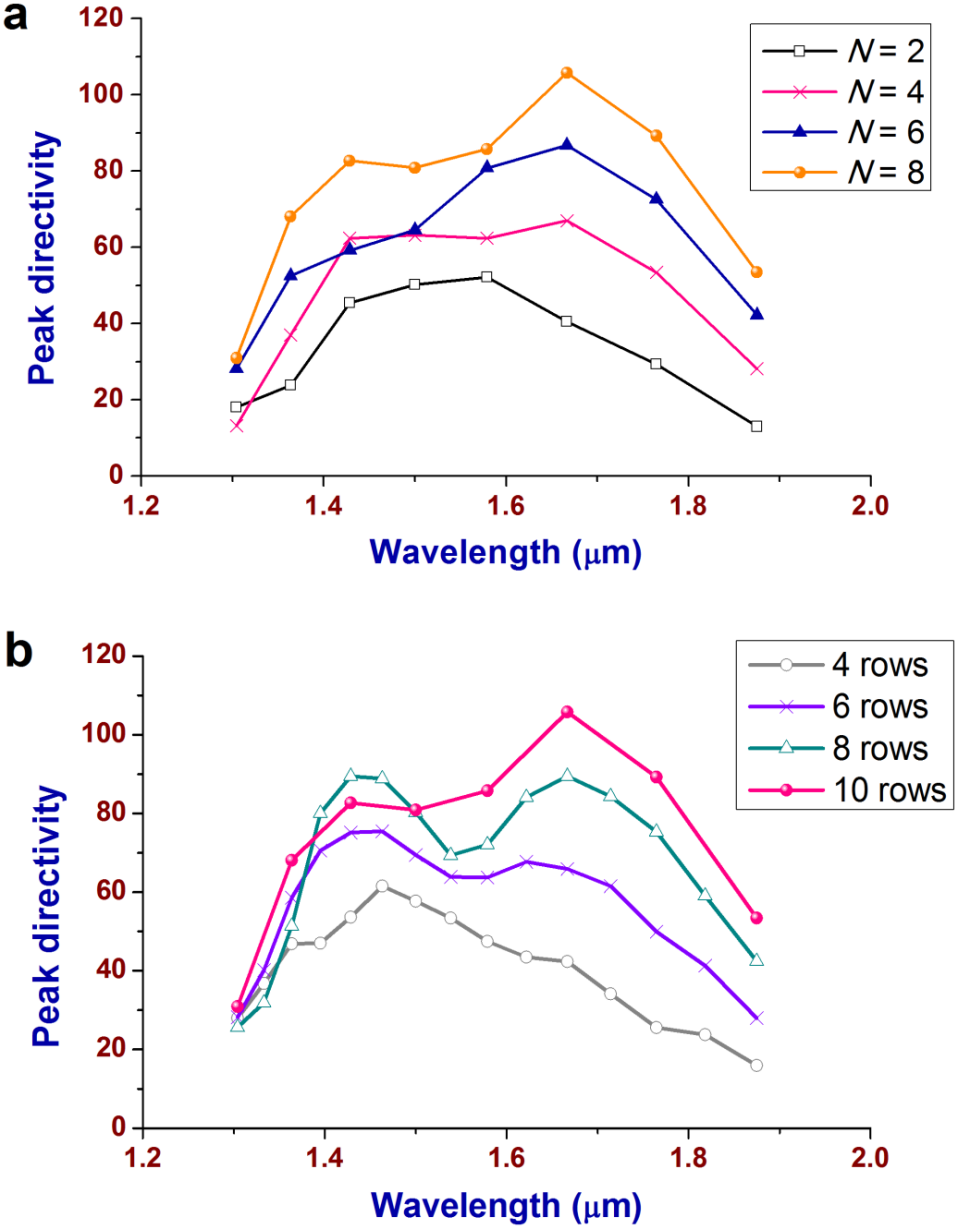
Two ways to enhance the peak directivity of the array. (a) Peak directivity of the nanopatch array versus *N*, the number of nanopatch antennas in one row. The peak directivity can be increased further by placing more nanopatch antennas in each row. However, as the nanopatch antennas are placed outside the “effective area” of the CWPWG mode, the peak directivity cannot be obviously improved any more. (b) Peak directivity of the nanopatch array versus the number of rows of nanopatch antennas. The peak directivity is gradually enhanced as the row number is increased from 4 to 10.

As the third merit of the proposed array, a more directive beam can be easily achieved by putting more nanopatch antennas in each row or arranging more rows of nanopatch antennas along the CWPWG. Figure 4a shows how the peak directivity is enhanced by increasing the number *N* of nanopatch antennas in each row. It is increased from 52.2 as *N* = 2 to 67 as *N* = 4, 86.7 as *N* = 6, and 105.7 as *N* = 8. The peak directivity should be theoretically enlarged by twice as *N* is doubled, according to array theory. However, the results deviate from the theoretical values because the excitation of the nanopatch antennas in one row is non-uniform and the mutual coupling between them also slightly changes the field distributions on and under the nanopatch antennas. Therefore, there is a tradeoff between the array peak directivity and the number of nanopatch antennas in each row. In this work, eight nanopatches are adopted in each row for easy of simulations, but more nanopatches are still effective for a higher directivity. Figure 4b presents how the number of rows of the nanopatch antennas exerts influence on the peak directivity. The maximum is gradually increased from 61.5 for 4 rows to 105.7 for 10 rows albeit at different wavelengths (See Supplementary Fig. S6 for comparisons of the directivity patterns). Actually, the peak directivity can also be further enhanced by placing more rows of nanopatch antennas along the CWPWG according to the given simple rules.

## Discussion

To the authors' knowledge, this work is the first one related to a nanoantenna array with a plasmonic waveguide, paving a way toward more practical optical IC applications. Except for the CWPWG, other plasmonic waveguides, e.g., nanowires^18^, dielectric loaded plasmonic waveguide^9^, and nanostrips^2^, can also be adopted for the nanopatch array design according to similar rules. It should be noted that if the antisymmetric mode of the CWPWG is applied, the distance *d_e_* of the nanopatch antennas on both sides of the waveguide should have a difference of π/*k_p_* (*k_p_* is the wave number of the guided mode) for coherent interference of the emitted optical waves within the xz plane.

The above results are investigated from the emission point of view, and the nanopatch array will demonstrate identical properties according to the reciprocity principle for an inverse process. Therefore, its potential applications can be extended from the optical ICs to lasers, optical communications, etc. For example, the PMMA in the proposed nanopatch array can be replaced by gain assistant materials like cadmium sulphide (CdS) to construct a highly directive plasmonic laser with low threshold intensity and extremely high output power density^38^. As another example, the high directivity property of the nanopatch array means the capability to collect more optical power from a given direction, which is critical for some applications in active optical devices, e.g., ultrafast photodiode with a contradiction between a high operating speed and compact dimensions^28^. Meanwhile, traditional photonic spectrometers are generally bulky, and thus some plasmonic structures like Yagi-Uda nanoantennas^39^ are proposed but still far from engineering applications. The proposed nanopatch array, with the ability to project different wavelengths into different angles, can be applied as a high performance optical spectrometer. Moreover, similar to the CPPs^5^,^6^, focused ion beam (FIB) milling techniques can be applied to fabricate the proposed nanopatch array, and other reported advanced fabrication techniques like nanoimprint lithography^40^ and electron beam lithography will be preferred. Additionally, the silver in the proposed array can also be replaced with gold or aluminum by taking the dispersive relation into account. Finally, for experimental purposes, two proposed nanopatch arrays can be placed back-to-back^22^, one for excitation and the other for emission, and its optical performances can be measured.

## Methods

### Theoretical Design

Beam direction of the proposed nanopatch antenna array can be calculated according to equation (3). 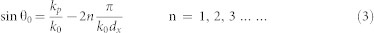
where *θ_0_* is the beam direction of the array, and *k_0_* is the free-space wave number. Given a designed wavelength, if the nanopatch antennas are placed close to the CWPWG by a distance of 2*n*π/*k_p_* for an efficient in-phase power coupling, the emitted power will be coherently superposed in the broadside direction. Furthermore, since the plasmonic waveguides including the CWPWG always present a dispersion curve below the light line, their wave number *k_p_* is always larger than free-space wave number *k_0_*. If *n* = 1 is chosen, the case in this work, the distance of neighboring nonapatch antennas should be less than one free-space wavelength, and then the nanopatch antenna array will not show grating lobes according to antenna array theory^37^. For other wavelengths, the beam directions can also be determined by equation (3). However, even if *n* ≥ 2 is selected, the grating lobes can still be controlled by redesigning the CWPWG for an appropriate *k_p_*.

### Numerical Calculation

The numerical results are obtained by using Ansoft High Frequency Structure Simulator (HFSS) based on the finite element method (FEM). The dispersive permittivity of silver is ε = ε′ – *j*ε″, where ε′ and ε″ are the real and imaginary parts, respectively, and 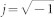. In the HFSS model, ε′ can be calculated according to the Drude model of silver, and the loss tangent tanδ = ε″/ε′, which is a negative value because of a negative ε′ but a positive ε″.

## Author Contributions

S.W.Q. conceived the idea and is responsible for theoretical design, structure construction and simulations. Z.P.N. gave constructive comments on the results. Both the authors contributed to the article.

## Supplementary Material

Supplementary Information Supplementary Information

Supplementary Information emission animation at 1.429μm

Supplementary Information emission animation at 1.5μm

Supplementary Information emission animation at 1.579μm

Supplementary Information emission animation at 1.667μm

Supplementary Information emission animation at 1.765μm
