# Supplementary material for: Plasmonic nanopatch array for optical integrated circuit applications
Source: Sci Rep. 2013 Nov 8;3:3172. doi: 10.1038/srep03172 (PMC3821018; doi:10.1038/srep03172)
Supplement: Supplementary Information [file srep03172-s1.pdf]

SUPPORTING INFORMATION

Plasmonic Nanopatch Array for Optical Integrated Circuit Applications

Shi-Wei Qu & Zai-Ping Nie

Table of Contents

S.1 PMMA Loaded Coupled Wedge Plasmonic Waveguide (CWPWG)…………………2

S.2 Impact of PMMA Thickness on Array Performances…………………………………3

S.3 Magnetic Field Distributions in Central Plane of the Plasmonic Array………………4

S.4 Array Efficiency ……………………………………………………………………6

S.5 3D Directivity Patterns………………………………………………………………...7

S.6 Enhancement of Peak Directivity ……………………………………………………. 9

S.7 CST Microwave Studio Results……………………………………………………10

S.8 Supplementary Animations………………………………………………………….. 14

List of Figures

Figure S1. Mode distribution and parametric studies of the CWPWG…………………………2

Figure S2. Influence and thickness of the PMMA layer……………………………………..3

Figure S3. Magnetic field distributions in the central plane……………………………………4

Figure S4. Simulated array efficiency at different wavelengths ………………………………6

Figure S5. Simulated 3D directivity patterns at different wavelengths ………………………..8

Figure S6. Directivity patterns of the array with different rows of nanopatch antennas at 1.5μm ……………………………………………………………………….. 9

Figure S7. Magnetic field distributions in the central plane obtained by the FIT……………11

Figure S8. Comparisons between the FEM and the FIT results …………………………….. 12

Figure S9. Simulated 3D directivity patterns at different wavelengths (FIT results) ………..13

S.1 PMMA Loaded Coupled Wedge Plasmonic Waveguide (CWPWG)

Electric field distributions of the operating mode are given in Figure S1a to show the mode in detail. Since the PMMA has a refractive index $n_{eff} = 1.49$, corresponding to a relative permittivity $\varepsilon_r = n_{eff}^2 \approx 2.22$, both the normal and the tangent components of magnetic fields are continuous according to the electromagnetic field boundary conditions, but the normal components of electric fields are related to the relative permittivity on both sides of the PMMA-air interface. Therefore, a discontinuity is observed at the PMMA-air interface. The mode size is mainly determined by the flare angle β of the V-groove, as shown in Figure S1b. When β is as small as 11°, two magnitude peaks of the magnetic fields are very close to each other and the two wedge modes are significantly coupled with each other, so the dip at the groove center is shallow, but the mode size is significantly enlarged. When the two wedge modes are less coupled as β increases, the dip becomes deeper and deeper.

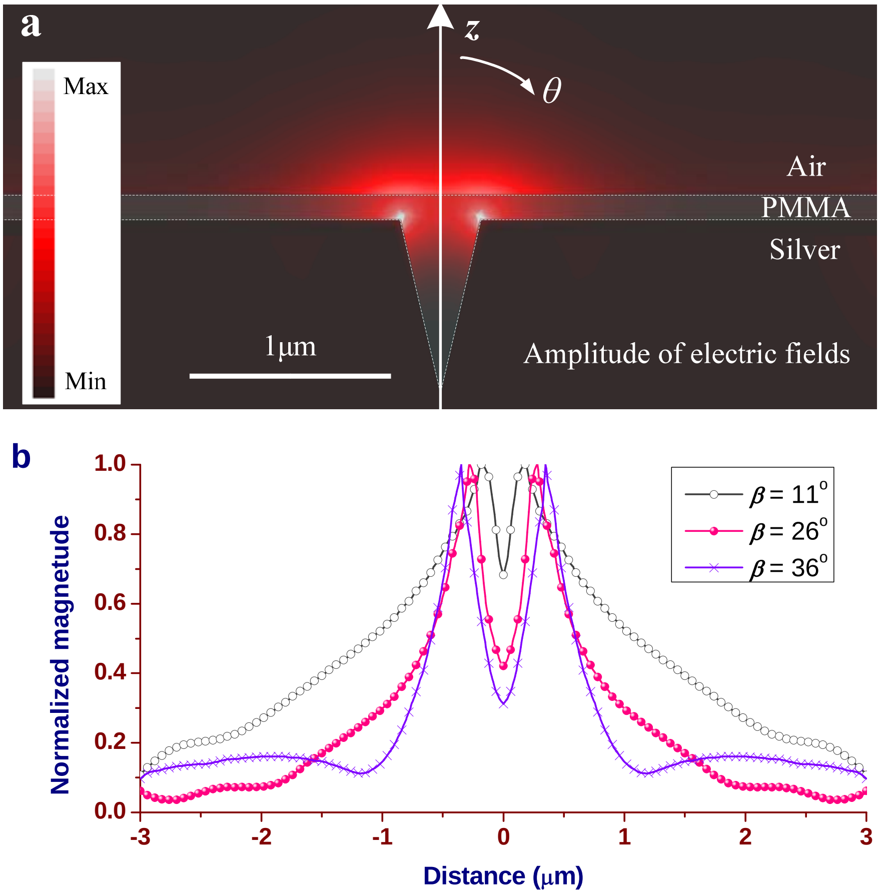

Figure S1. Mode distribution and parametric studies of the CWPWG. (a) Electric field distributions within the cross section of the CWPWG. The optical power is concentrated close to the aperture of the groove. The mode distributions are changed by the loaded PMMA. The dielectric-loading effect are investigated previously (Karalis, A., *et al.* Surface-plasmon-assisted guiding of broadband slow and subwavelength light in air. *Phys. Rev. Lett.* 95, 063901, 2005.), which can also provide another way to control the mode distributions of the CWPWG. (b) Normalized magnitude of magnetic fields along a referenced line, which is orthogonal to the CWPWG central axis and 50nm above the silver film.

S.2 Impact of PMMA Thickness on Array Performances

Thickness *t* of the PMMA layer will exert influence on the plasmonic array performance. As shown in Figure S2a, the beam direction is shifted from -13° as *t* = 100nm to -7° as *t* = 200nm at 1.667μm, mainly due to dependence of the beam direction on the wave number of the propagating mode in the CWPWG which is sensitive to thickness *t*. At the same time, the more shifted beam by a thinner PMMA layer will also cause a reduction of array effective aperture, consequently resulting in a lower peak directivity, as shown in Figure 2b. Moreover, a thinner PMMA layer will lead to more intense electric or magnetic fields under the nanopatches and higher quality factor of the cavity formed by the nanopatches and the silver film, narrowing the operating wavelength range.

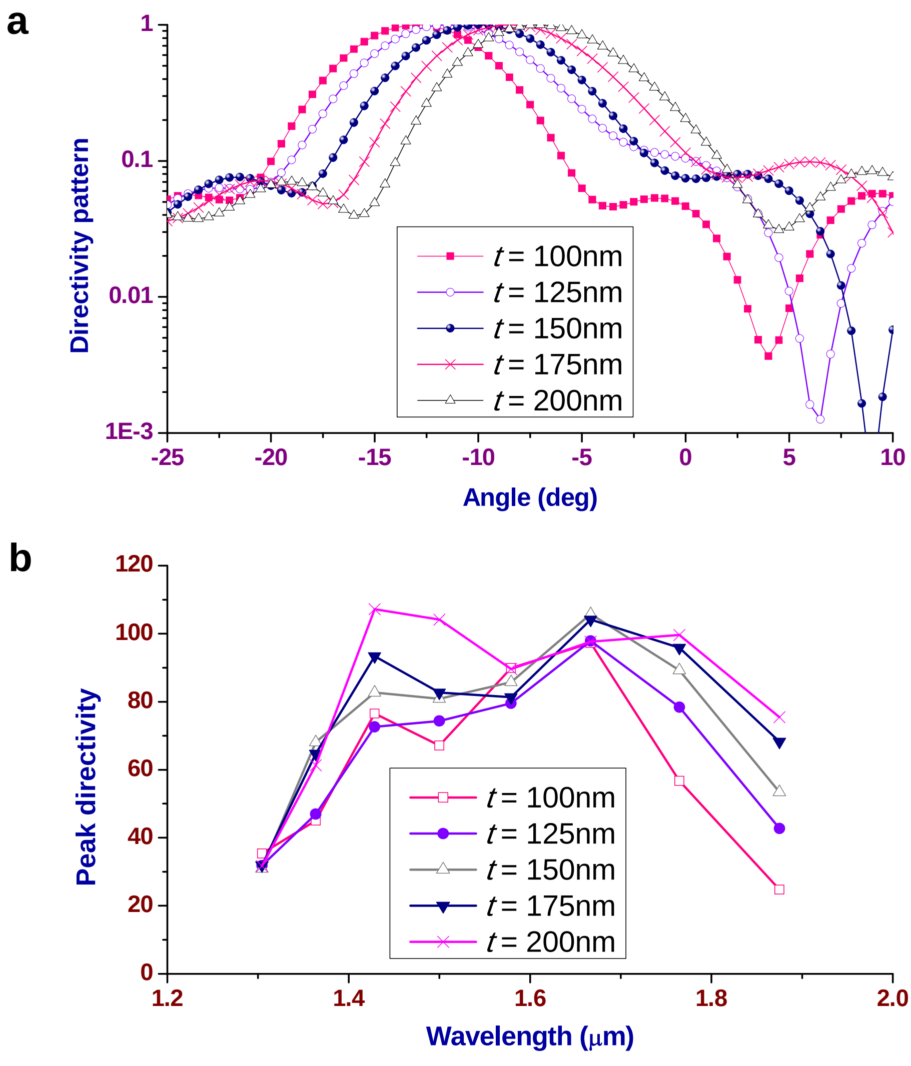

Figure S2. Influence and thickness of the PMMA layer. (a) Beam direction at 1.667μm versus different thickness *t* of the PMMA layer. (b) Peak directivity of the plasmonic nanopatch array versus different PMMA thickness *t*.

S.3 Magnetic Field Distributions in Central Plane of the Plasmonic Array

Magnetic near field distributions of the proposed nanopatch array at 1.765, 1.667, 1.579, 1.5, and 1.429μm are shown in Figure S3. For clarity, only the near fields close to the last six nanopatch antennas are given albeit 10 in the proposed array.

Clearly, the optical waves emitted by the nanopatch antennas create a plane with coherent interference in front of the array which determines the emission direction of the array. Meanwhile, more optical power is reflected by the shorted termination as the operating wavelength is increased. Without the termination, larger parasitical beams will occur, which explains its functions in improving the directivity patterns and the spectral width. The animations of near-field distributions at all wavelengths can also be found in the supplementary materials.

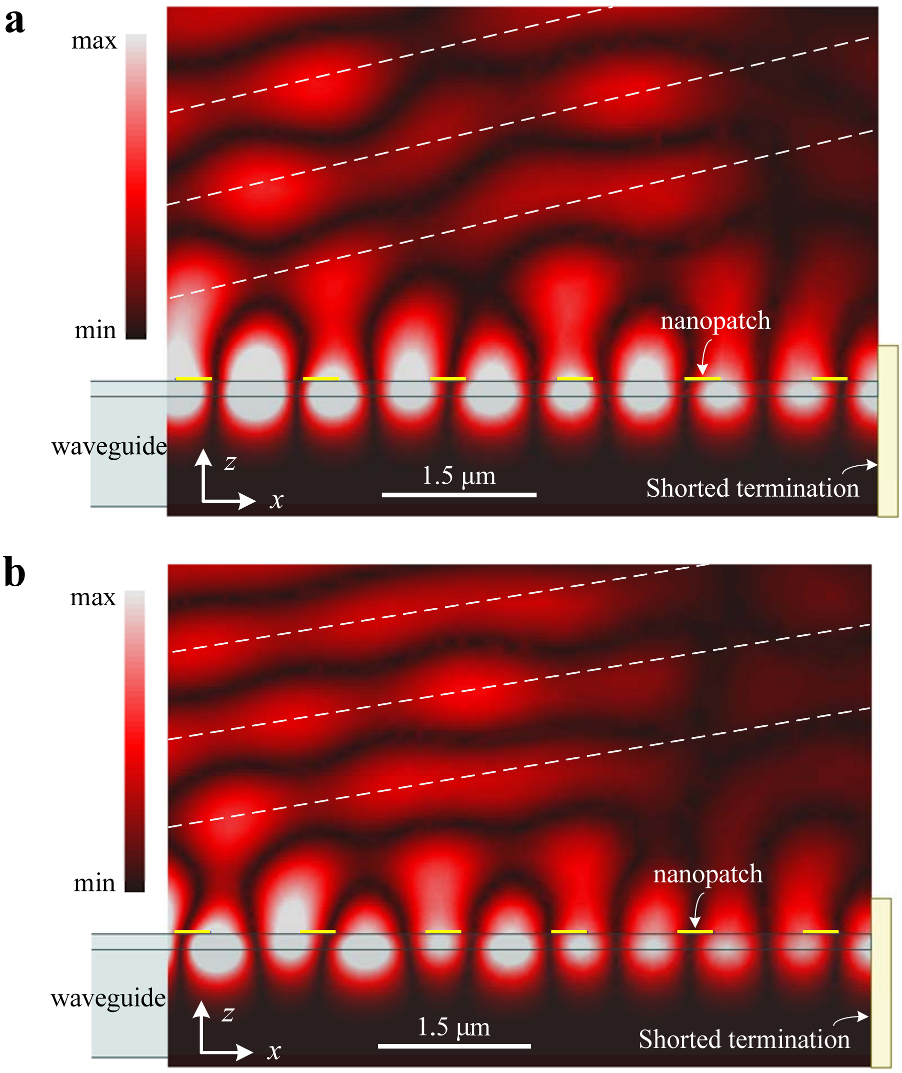

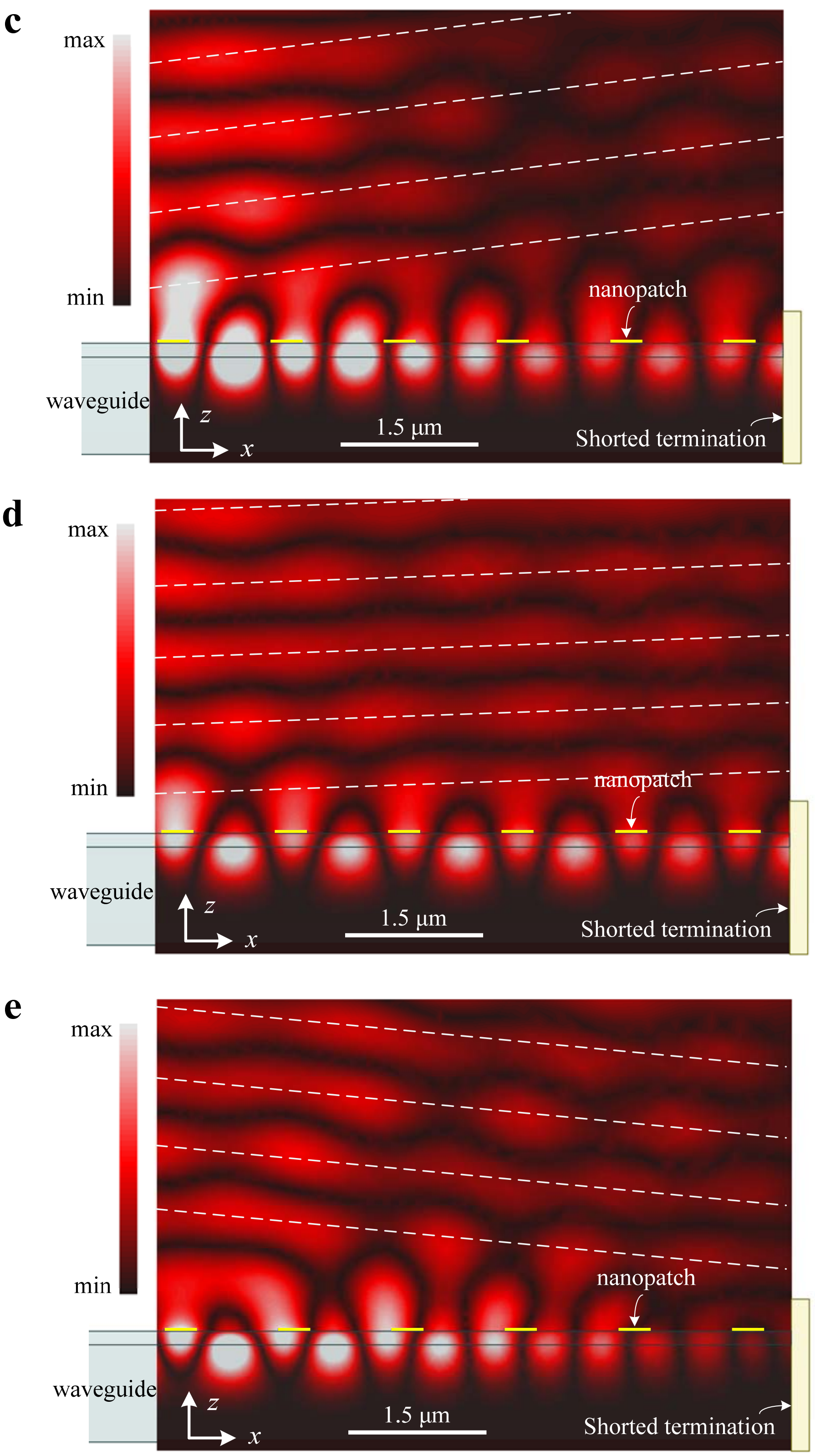

Figure S3. Magnetic field distributions in the central plane. (**a**) 1.765μm. (**b**) 1.667μm. (**c**) 1.579μm. (**d**) 1.5μm. (**e**) 1.429μm. The white dashed lines show the coherent interference wavefront of the optical waves emitted by the nanopatch antennas and direction of the emitted beam by the nanopatch array.

S.4 Array Efficiency

Array efficiency, defined by the whole emitted power into free space over the accepted power by the array, is used to measure the array ability to transform the guided plasmonic waves into free-space optical waves. As shown in Figure S4, the nanopatch array presents the highest array efficiency of 75.4% at around 1.579μm.

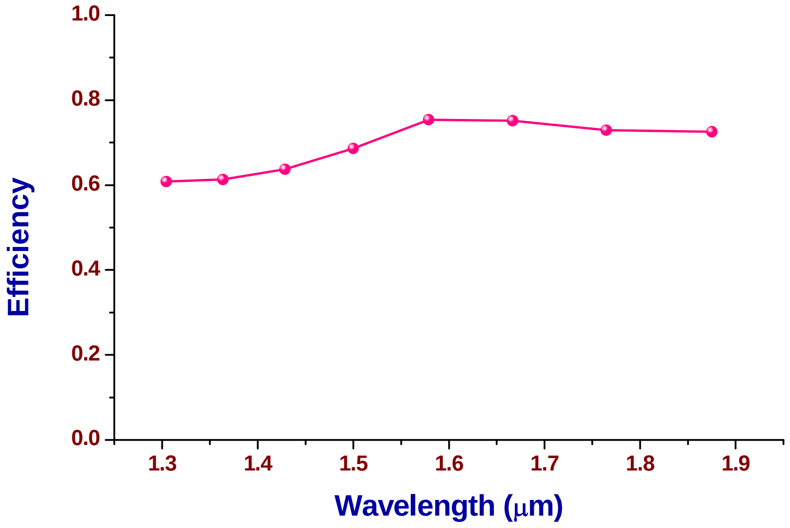

Figure S4. Simulated array efficiency at different wavelengths. Thanks to the less confined mode of the CWPWG and high efficiency of the nanopatch antennas, the array efficiency is in a range of 61% ~ 75.4% over the whole operating spectral width. Drop of the array efficiency at shorter wavelengths is caused by both the detuned properties of the nanopatch antennas and the larger dissipation of silver according to the Drude model, while the drop at longer wavelengths is mainly caused by the former.

S.5 3D Directivity Patterns

To present more details, 3D directivity patterns at wavelengths of 1.765, 1.667, 1.579, 1.5 and 1.429μm are shown in Figure S5. It can be seen that there is only one main beam at each wavelength, the parasitical beams are relatively small, and the backward emission of the proposed array at all wavelengths is quite smaller relative to the main emission beam. All of these properties mean that most of the optical power are emitted in a solid angle around the main beam. Therefore, the 2D directional patterns in Figure 3a of the main content can present typical redirection properties of emission.

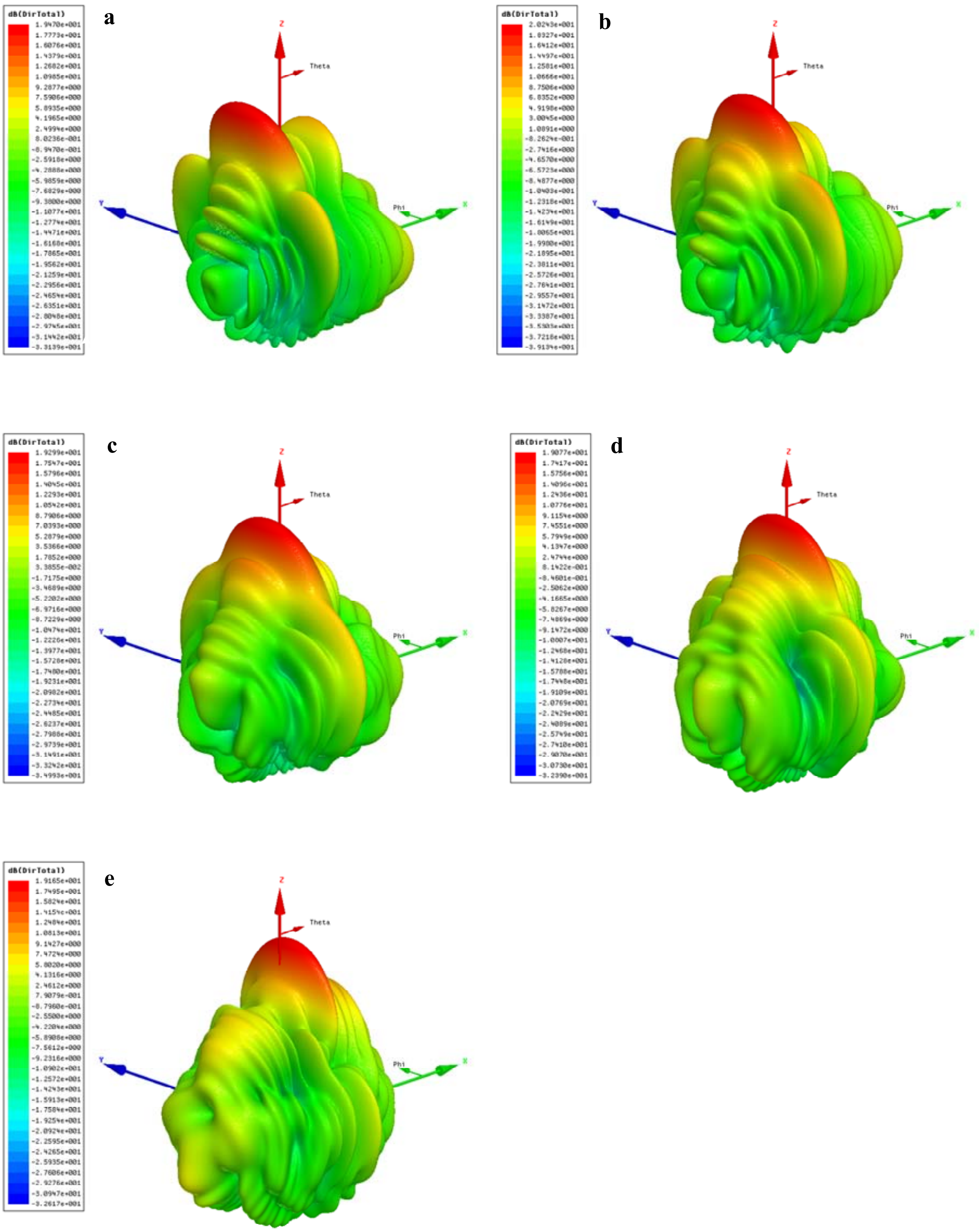

Figure S5. Simulated 3D directivity patterns at different wavelengths. (**a**) 1.765µm. (**b**) 1.667µm. (**c**) 1.579µm. (**d**) 1.5µm. (**e**) 1.429µm. Logarithm scale is adopted, i.e., in dB, to clearly show the details of directivity patterns. The main beam is gradually shifted from -x to +x direction as the wavelength decreases from 1.765 to 1.429µm. The parasitical beams are around 0.1 times of the main beam or even smaller, i.e., 10dB lower than the main beam.

S.6 Enhancement of Peak Directivity

Directivity of the nanopatch array can be easily enhanced by adding more nanopatch antennas in each row or by placing more rows of nanopatch antennas, which is one of the advantages of the proposed nanopatch array. As complementary results to Figure 4 in the main context, Figure S6 shows the directivity patterns of the proposed array with different rows of nanopatch antennas.

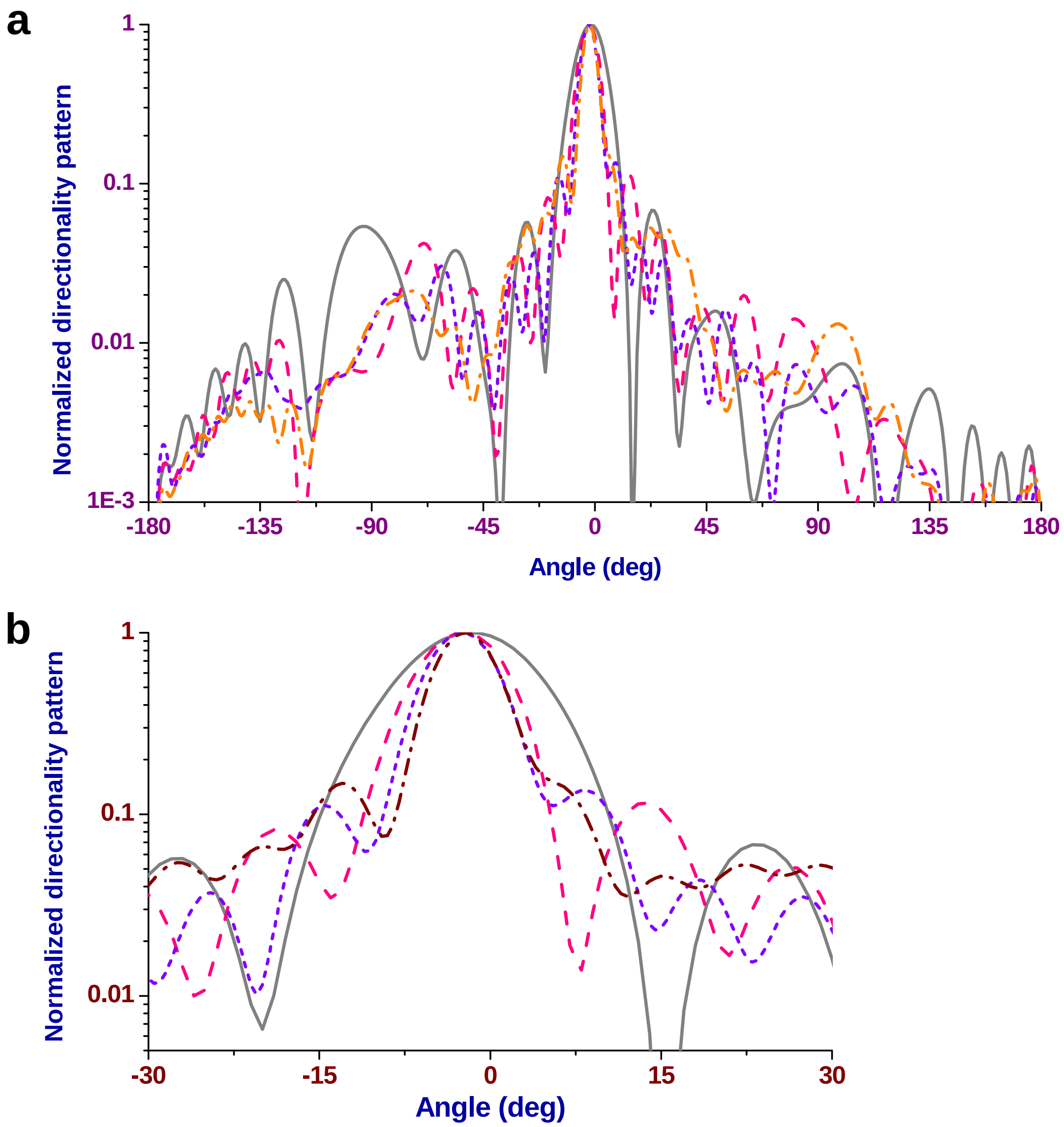

Figure S6 | Directivity patterns of the array with different rows of nanopatch antennas at 1.5μm. (**a**) Broad angle view and (**b**) zoom view of the directivity patterns. Gray solid curve: 4 rows, pink long dashed curve: 6 rows, violet dotted curve: 8 rows, wine dash-dotted curve: 10 rows. For all cases, the parasitical beams are quite small. Clearly for the array with less rows of nanopatch antennas, the beam width is broader than the one with more rows due to smaller directivity.

S.7 CST Microwave Studio Results

To verify the resutls obtained by Ansoft High Frequency Structure Simulation (HFSS) based on the finite element method (FEM), the CST Microwave Studio based on the finite integration technique (FIT) is used to resimulate the proposed nanopatch array. Figure S7 shows the near-field distributions of the array at 1.765, 1.667, 1.579, 1.5 and 1.429μm by using the FIT. Obviously, there is no noticeable differences from the FEM results in term of emission direction and relative magnitude.

Figure S8 presents comparisons of the directivity patterns and peak directivity obtained by the FEM and FIT. Obviously, the xz- and yz-plane directivity patterns of the principle and cross polarizations and beam positions by the FIT are quite similar to the FEM ones in the angle range of the main beam. Only a little difference can be found outside the main beam. The spectral width in terms of the peak directivity obtained by the FIT is slightly red-shifted relative to the FEM results. The small differences in terms of directivity patterns and peak directivity are mainly caused by the different meshes of two methods, i.e., triangular meshes in the FEM but hexahedral ones in the FIT.

Figure S9 gives the simulated 3D directivity patterns at 1.765, 1.667, 1.579, 1.5 and 1.429μm by using the FIT. Compared to those in Figure S5, there are only small differences in terms of the parasitic beams. The above comparisons between the FIT and the FEM results prove the correctness and validity of the results in the main context.

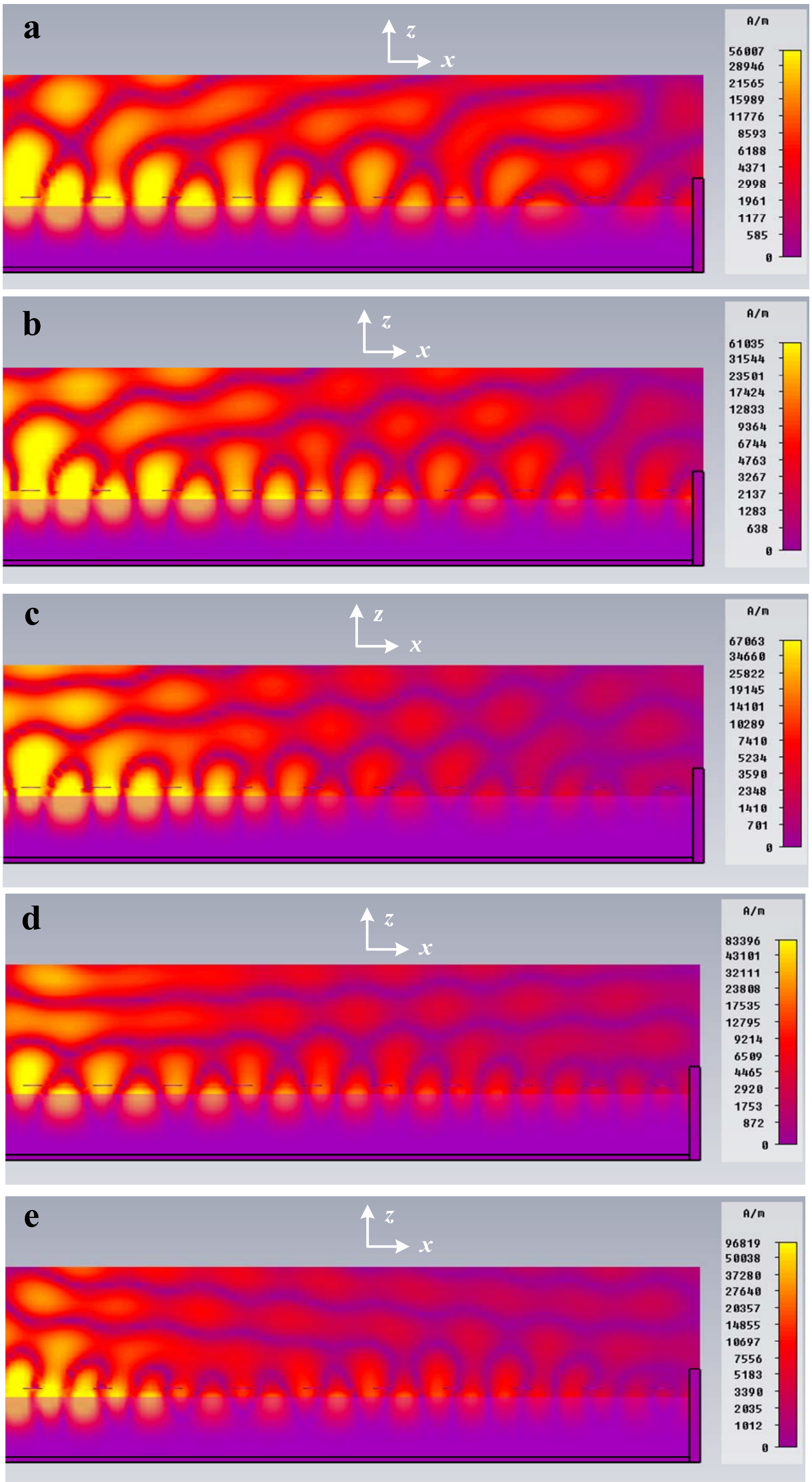

Figure S7. Magnetic field distributions in the central plane obtained by the FIT. (**a**) 1.765µm. (**b**) 1.667µm. (**c**) 1.579µm. (**d**) 1.5µm. (**e**) 1.429µm. The coherent interference wavefronts are quite similar to the FEM results.

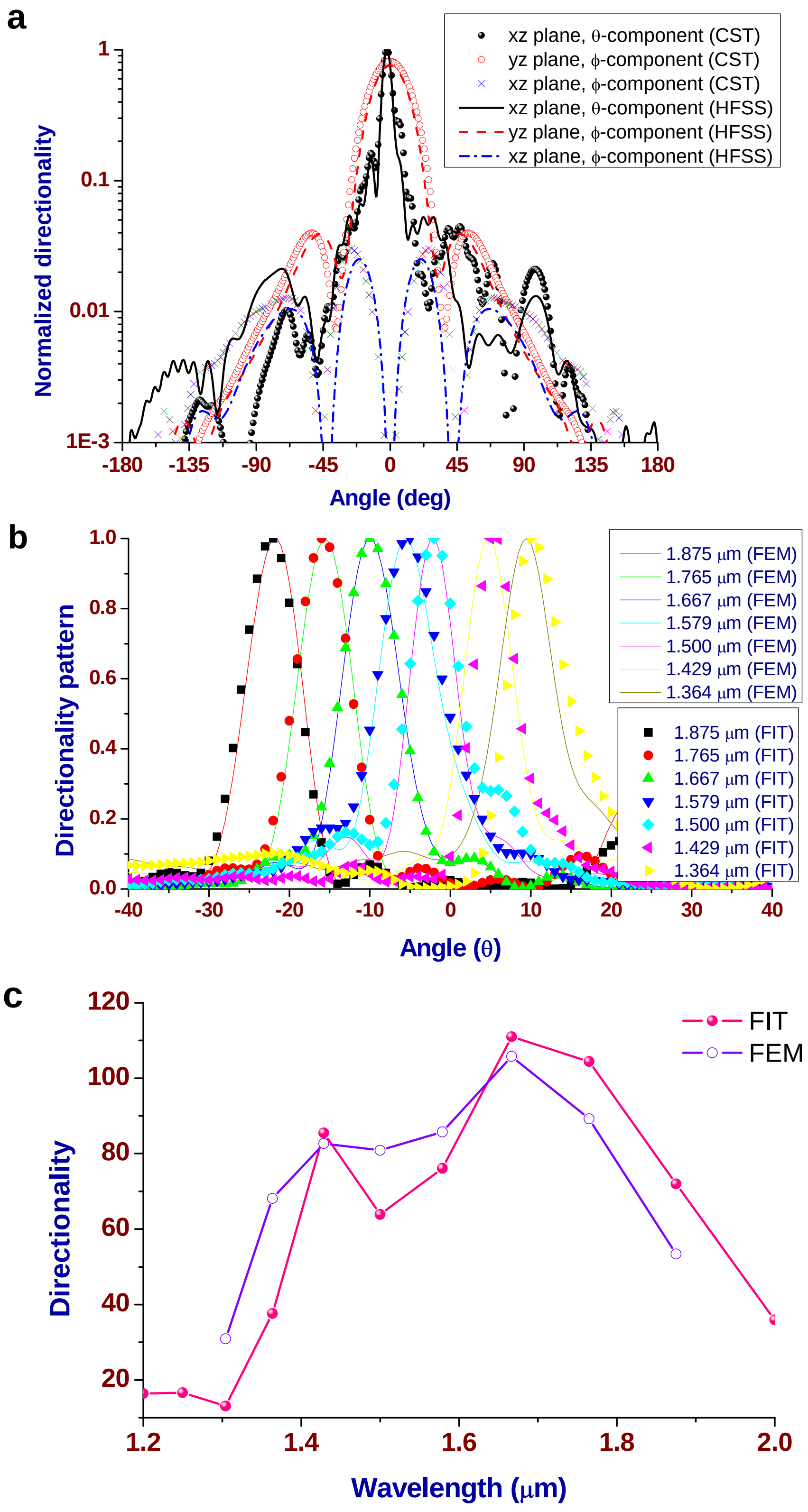

Figure S8. Comparisons between the FEM and the FIT results. (**a**) Comparisons of directivity patterns in the xz and yz planes at 1.5μm. (**b**) Comparisons of the normalized directivity patterns at different wavelengths. (**c**) Comparisons of peak directivity.

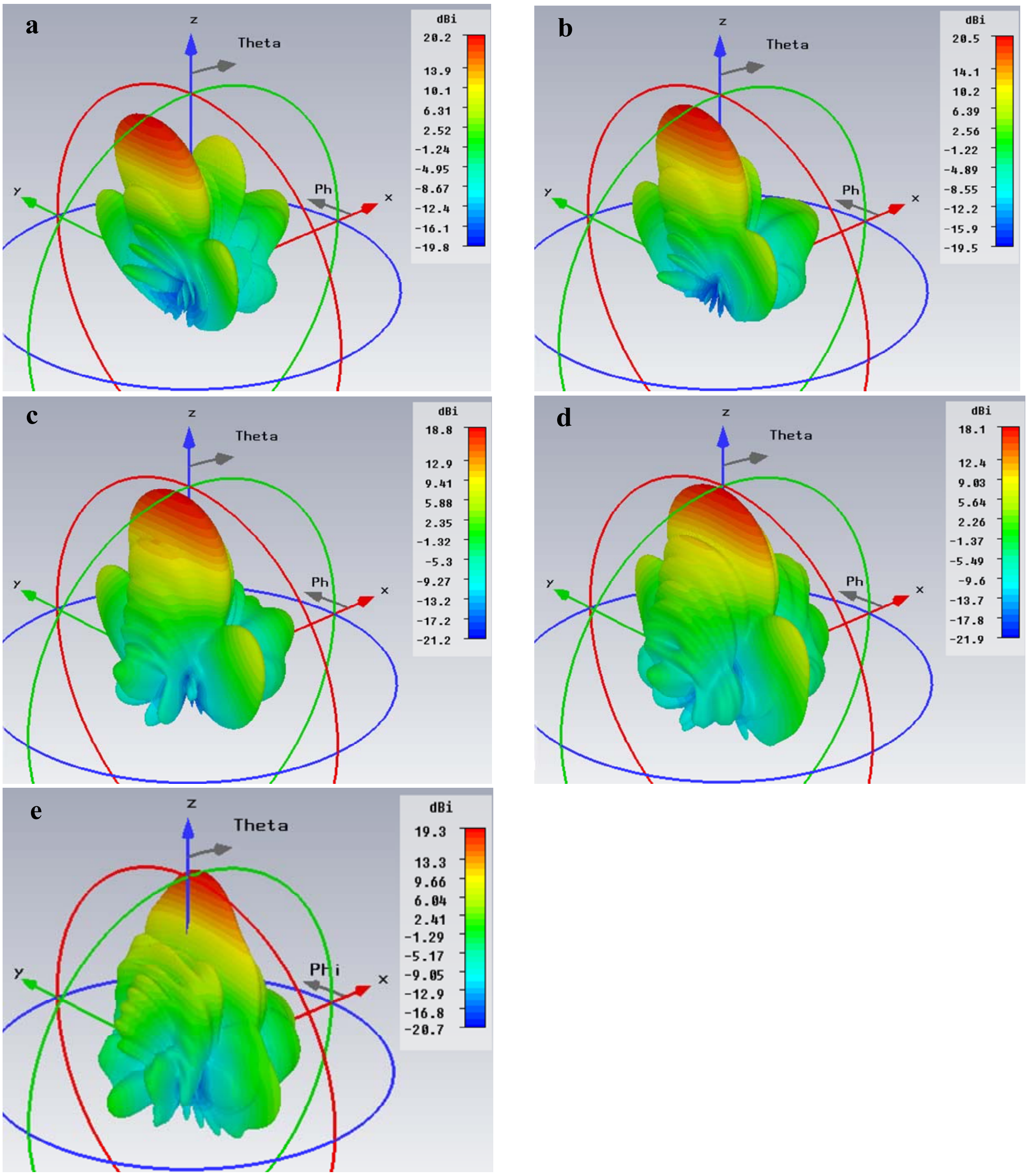

Figure S9. Simulated 3D directivity patterns at different wavelengths (FIT results). (**a**) 1.765µm. (**b**) 1.667µm. (**c**) 1.579µm. (**d**) 1.5µm. (**e**) 1.429µm. Logarithm scale is adopted (in dB), also to clearly show the details of directivity patterns. The results obtained by the FIT also shows reasonable agreements with those by the FEM.

S.8 Supplementary Animations

Animations of the magnetic near fields at 1.429, 1.5, 1.579, 1.667, and 1.765μm are given as supplementary information.
