# Supplementary figures and images for: Plasmonic nanopatch array for optical integrated circuit applications
Source: Sci Rep. 2013 Nov 8;3:3172. doi: 10.1038/srep03172 (PMC3821018; doi:10.1038/srep03172)

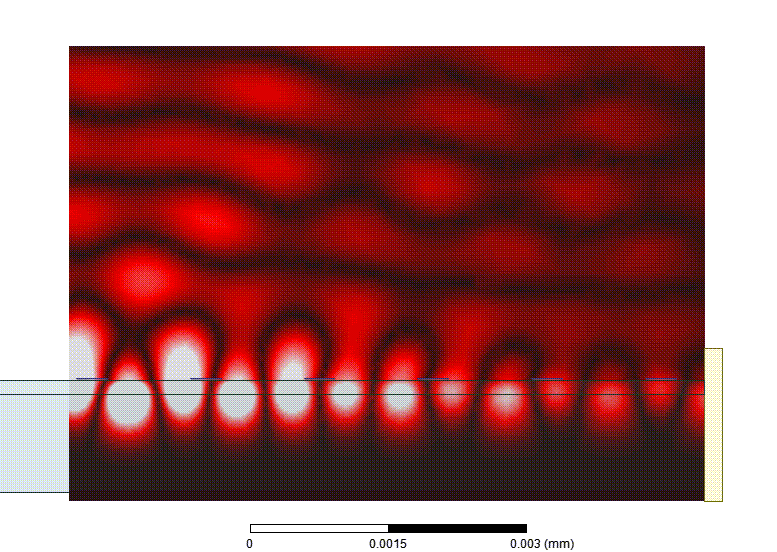

Supplement: Supplementary Information — emission animation at 1.429μm [file srep03172-s2.gif]

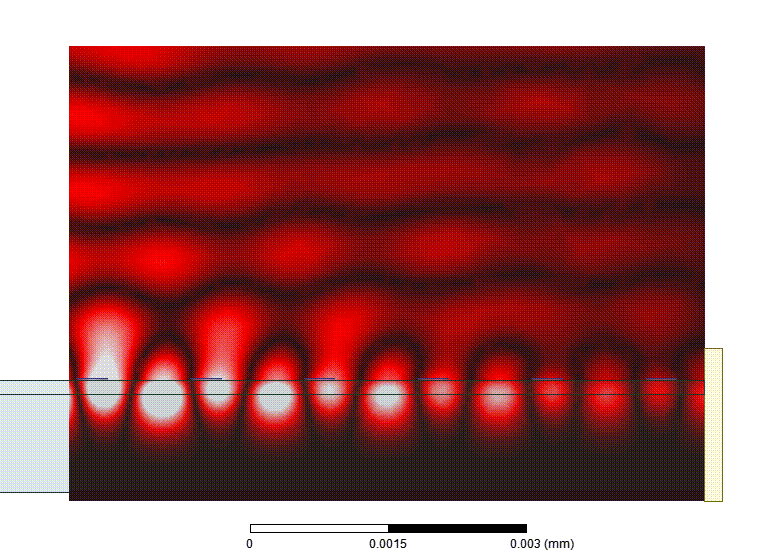

Supplement: Supplementary Information — emission animation at 1.5μm [file srep03172-s3.gif]

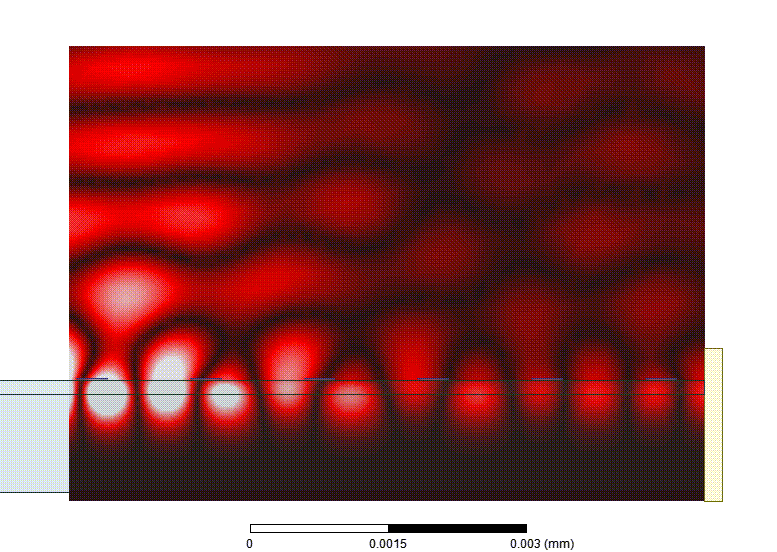

Supplement: Supplementary Information — emission animation at 1.579μm [file srep03172-s4.gif]

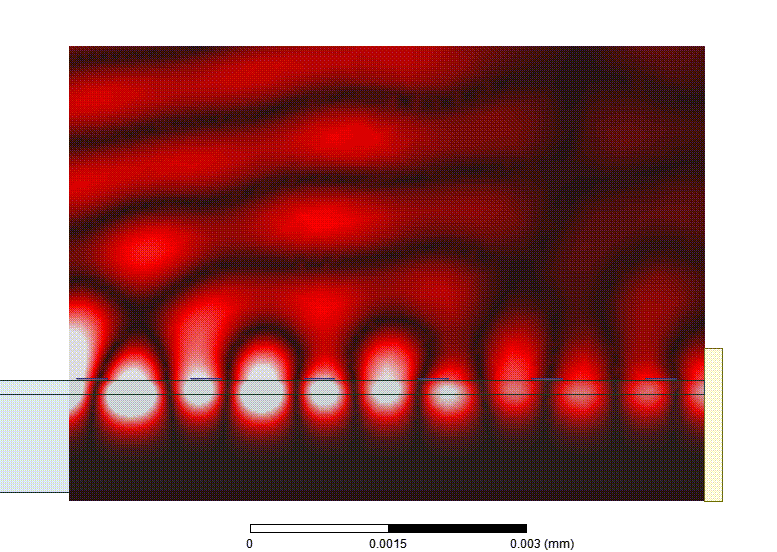

Supplement: Supplementary Information — emission animation at 1.667μm [file srep03172-s5.gif]

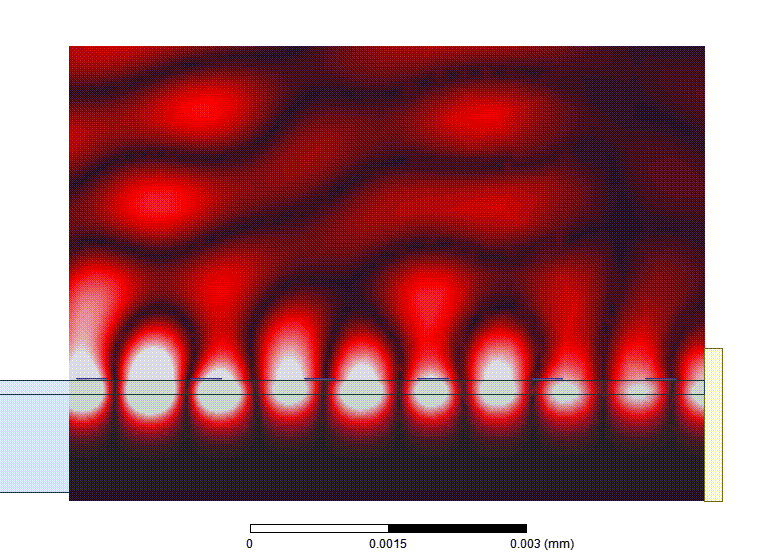

Supplement: Supplementary Information — emission animation at 1.765μm [file srep03172-s6.gif]
